# Attenuated titin protein expression is associated with advanced stages of ovarian cancer

**DOI:** 10.1016/j.omton.2025.200932

**Published:** 2025-01-14

**Authors:** Harvey Sharma, Jaskaran Aujla, Asad Nawaz, Thabet Khasawneh, Ayesha Alvero, Robert T. Morris, Asma Basha, Laila Tutunji, Toshima Z. Parris, Khalil Helou, Ghassan M. Saed

**Affiliations:** 1 Department of Obstetrics and Gynecology, Wayne State University School of Medicine, Detroit, MI 48201, USA; 2 Department of Gynecologic Oncology, Karmanos Cancer Institute, Detroit, MI 48201, USA; 3 Department of Obstetrics and Gynecology, University of Jordan School of Medicine, 226 Queen Rania Al Abdullah Street, Amman 11942, Jordan; 4 Department of Oncology, Institute of Clinical Sciences, Sahlgrenska Academy, University of Gothenburg, Box 711 405 30, Gothenburg, Sweden; 5 Sahlgrenska Center for Cancer Research, Sahlgrenska Academy, University of Gothenburg, 41685 Gothenburg, Sweden

**Keywords:** MT: Regular Issue, attenuated titin expression, chemoresistance, ovarian cancer, therapeutic marker, titin mutations

## Abstract

This study explores the role of titin, a giant muscle protein, in the progression of epithelial ovarian cancer (EOC). We examined titin levels in tissues and sera from EOC patients across stages I–IV and in chemoresistant EOC cells. Tissue samples underwent immunohistochemistry, and serum titin levels were measured using ELISA. Quantitative real-time PCR analyzed titin mRNA in cell lines, including chemosensitive, chemoresistant, and normal ovarian cells. Notably, elevated titin levels were detected in 90.9% of stage I tissues compared to only 14.3% of stage III and IV tissues. Serum titin levels were consistently decreased across all stages relative to healthy controls, with a gradual decrease in expression from stages I to IV. Additionally, titin levels were significantly higher in normal ovarian epithelial cells compared to both chemosensitive and chemoresistant EOC cells, albeit significantly higher in chemosensitive than chemoresistant cells. These findings suggest the possible role of decreased titin levels as a marker for therapeutic intervention, particularly in advanced-stage and chemoresistant EOC. Further elucidation of the mechanisms underlying attenuated titin expression holds promise for advancing our understanding of ovarian cancer pathogenesis.

## Graphical abstract

**Figure:**
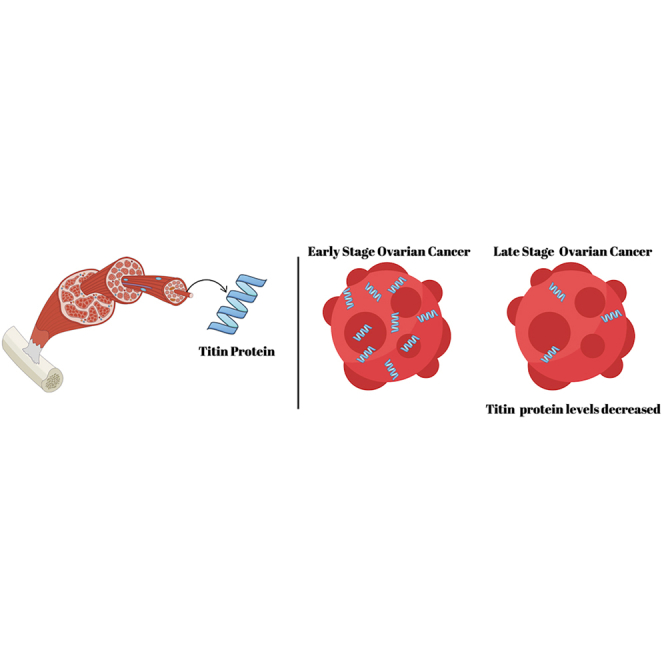

## Teaser

Saed and colleagues validate the role of titin in epithelial ovarian cancer (EOC). They found elevated titin levels in 90.9% of stage I tissues, decreasing in later stages, and chemoresistant cells. Serum titin levels also decrease with cancer progression. Reduced titin may serve as a marker for therapeutic intervention, especially in advanced-stage EOC.

## Introduction

Epithelial ovarian cancer (EOC) is the deadliest among the gynecologic cancers.^1^ Due to a lack of effective early detection methods, EOC often eludes diagnosis until an advanced stage, culminating in a mere 5-year survival rate of 30.2%.^1^ The current standard approach to managing EOC involves a combination of cytoreductive surgery and chemotherapy. Despite achieving initial complete clinical responses, a significant portion of patients ranging from 50% to 80% experience relapse with chemoresistant disease.^2^ This chemoresistance greatly hampers the effectiveness of subsequent treatments.

Titin, also known as connectin, encoded by the *TTN* gene, is a giant sarcomeric protein that interacts with actin and myosin, playing pivotal roles in the structural, mechanical, and regulatory functions within the sarcomere of striated muscle.^3^ Comprising 38,138 amino acids and boasting a mass of 4.2 MDa, titin belongs to the CAMK Ser/Thr protein kinase superfamily.^4^^,^^5^ The fragmentation of titin protein into the urine titin N-terminal fragment has emerged as a promising biomarker for muscle damage,^6^ with recent investigations exploring its implications in cancer development and progression.^7^^,^^8^^,^^9^^,^^10^^,^^11^ The TTN gene is known to acquire an abundance of somatic driver mutations strongly associated with several primary cancers.^12^ Notably, OC exhibits one of the highest rates of somatic titin mutations.^13^ Furthermore, the TTN gene produces several titin protein isoforms of different sizes (∼3–4 MDa) resulting from alternative splicing.^14^ The isoforms include (1) N2BA NM_001256850, which encodes a major cardiac TTN isoform and comprises the N2A and N2B exons and the PEVK region; (2) IC NM_001267550.2, the longest isoform as it contains all possible in-frame coding exons; this TTN isoform (IC) represents an inferred complete model of 363 exons and encodes a protein of 35,991 amino acids; (3) N2.An NM_133378.4, a transcript variant that encodes the predominant TTN isoform in skeletal muscle; this variant lacks the N2B region, while maintaining the PEVK region; (4) novex-1 or novex-1/N2B NM_133432.3, a transcript variant that encodes the minor cardiac muscle isoform, which is nearly identical to the major cardiac isoform N2-B but contains a unique stretch of 125 amino acids in the I-band region; (5) novex-2 or novex-1/N2B NM_133437.4, a transcript variant that encodes the minor cardiac and skeletal muscle isoform, which is nearly identical to the major cardiac isoform N2-B but contains a unique stretch of 192 amino acids in the I-band region; and (6) novex-3 NM_133379.5, a transcript variant that contains a stop codon and poly(A) tail signal. Moreover, the TTN isoform is expressed in all striated muscle, extending from the Z-disc region.^15^

Changes in titin expression in breast, lung, colorectal, and gastrointestinal cancers have been strong indicators of its role in cancer development and progression.^7^^,^^8^^,^^9^^,^^10^^,^^11^ The objective of the present study is to evaluate titin expression in tissues and sera of EOC patients from various stages as well as in chemoresistant EOC cells. Here, we show a significant decrease in titin expression in the tissues and sera from advanced-stage ovarian cancer compared to early-stage patients and healthy controls. Similarly, titin expression in EOC cells was downregulated relative to normal ovarian surface epithelial cells. Notably, titin exhibited lower expression levels in docetaxel- and cisplatin-resistant EOC cells compared to their chemosensitive counterparts. These findings illuminate the possibility of titin as a biomarker for EOC, opening avenues for further exploration.

## Results

### Titin is detected in ovarian tumors and downregulated during disease progression

**Figure 1:**
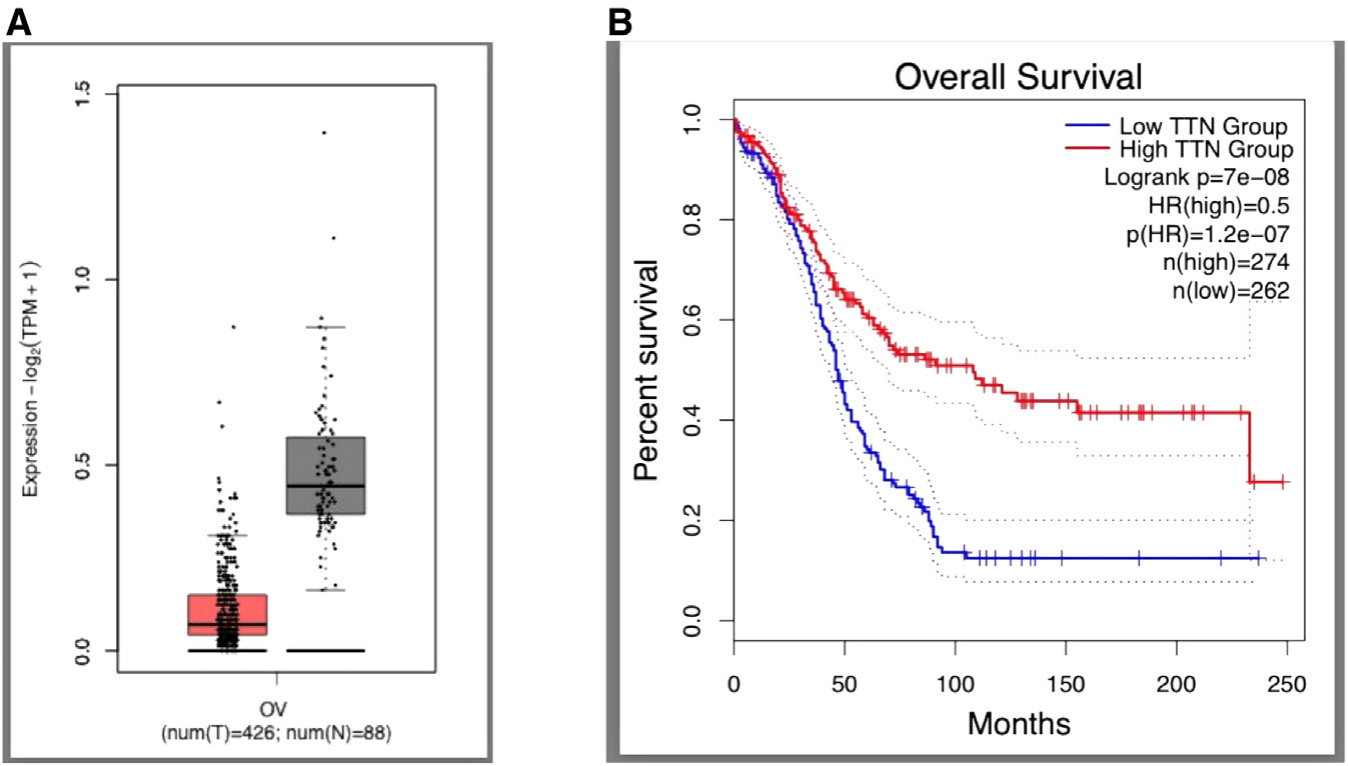
TTN is downregulated in ovarian cancer tissue and is associated with poor prognosis According to the GEPIA database, (A) TTN is downregulated in ovarian cancer (OC) tissue, ∗*p* < 0.05, and (B) low expression of TTN is associated with poor prognosis. Cutoff-high, 50%, and cutoff-low, 50%. Solid lines indicate survival curves; dotted lines indicate 95% confidence interval. GEPIA, Gene Expression Profiling Interactive Analysis; HR, hazard ratio; N, normal; TPM, transcripts per million; TTN, T, tumor.

**Figure 2:**
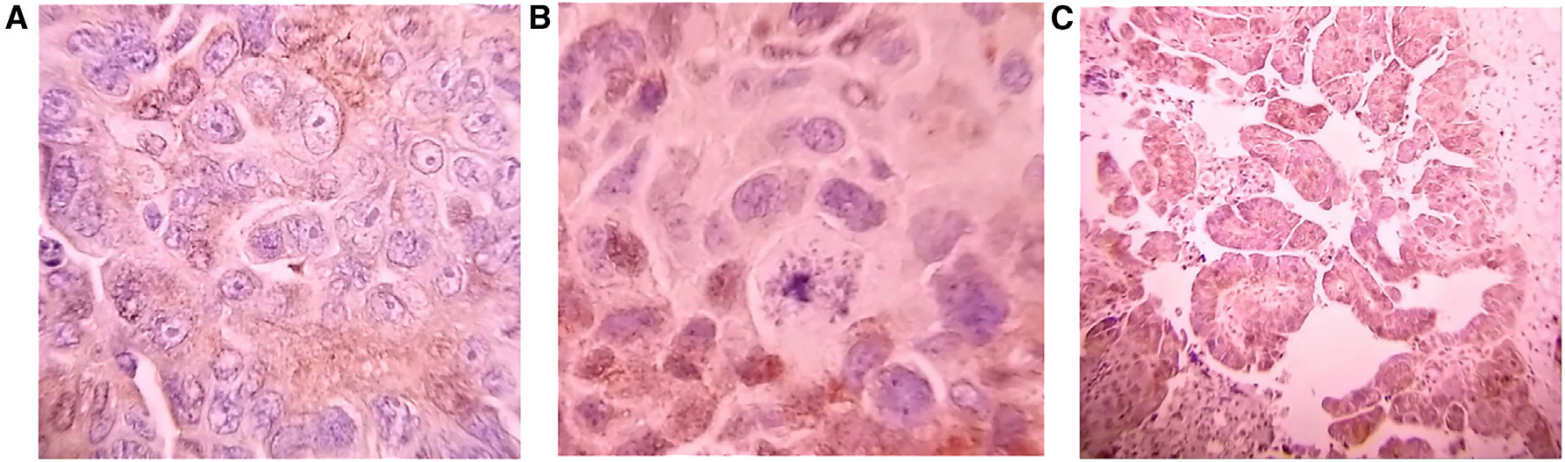
IHC staining of OC tissues with titin-specific antibody A representative IHC staining for titin in stage I OC tissues, score 3 reflecting high staining (A) and in stage IV OC tissues, score 1 reflecting low staining (B). A negative control where titin primary antibody was omitted, score 0 reflecting no staining (C). Images were captured at 40× magnification using a Zeiss microscope. Representative images of score 2 staining were not obtained.

The Gene Expression Profiling Interactive Analysis (GEPIA) database was used to examine TTN expression in ovarian cancer tissues (Figure 1). Expression of TTN was decreased in OC compared with that in normal ovarian tissue (Figure 1). To confirm these findings in our patient population, we performed immunohistochemistry (IHC) and found cytoplasmic titin staining in all the examined OC tissues (Figure 2).^16^ Next, we investigated whether the level of titin correlates with EOC disease stage. We found the intensity of titin staining to be inversely correlated with disease stage (Table 1). High levels of titin were detected in 90.9% of stage I EOC patients, and the remaining 9.1% of patients had moderate titin expression (Table 1). Of the stage II patients, 76.5% had high levels of titin, 17.6% had moderate levels, and 5.8% had low levels. Among the stage III patients, high titin expression was observed in only 14.3%, with moderate titin expression in 81.0%, and low titin expression in 4.8%. High levels of titin expression were detected in only 14.3% of stage IV patients, and medium and low levels were observed in 77.4% and 8.6%, respectively. There was a significant reduction in titin expression when comparing stage I to all the other stages (II–IV; *p* < 0.05). Similarly, there was a significant decrease in titin expression when comparing stage II to stages III and IV (*p* < 0.05). However, there was no significant difference in titin expression between stages III and IV.

**Table 1:** IHC cytoplasmic staining of titin protein in all OC tissues among different stages

Stage	High (%)	Medium (%)	Low (%)	*p*
I	20 (90.9)	2 (9.1)	0 (0)	−
II	13 (76.5)	3 (17.65)	1 (5.88)	0.001
III	3 (14.3)	17 (81.0)	1 (4.8)	−
IV	5 (14.3)	27 (77.1)	3 (8.6)	0.003

### Circulating titin levels are significantly decreased during EOC disease progression

**Figure 3:**
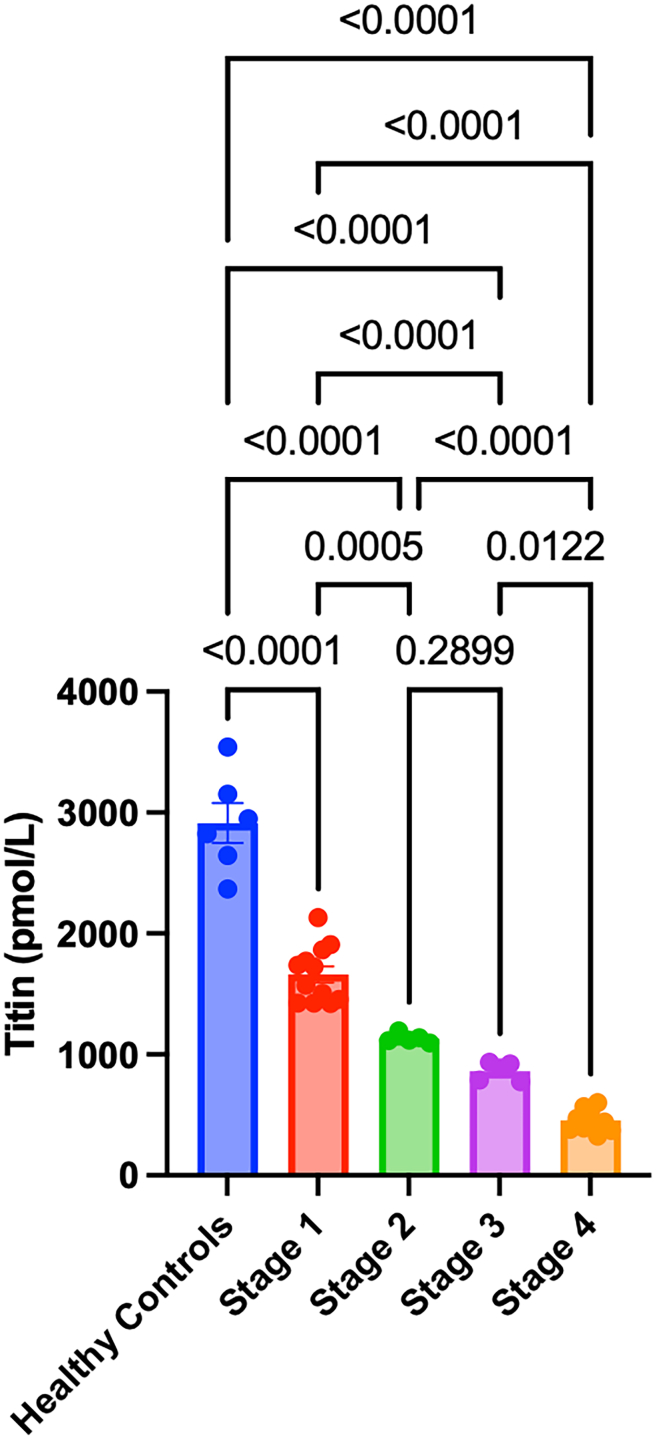
Titin protein concentrations in sera of five different patient groups Healthy control (*n* = 6), stage I (*n* = 11), stage II (*n* = 5), stage III (*n* = 5), and stage IV (*n* = 10). Titin protein concentration was quantitated in all samples by ELISA in picomoles per liter.

Given that we observed lower titin expression in advanced-stage EOC, we next determined whether titin can be detected in patient blood samples and the correlation of circulating titin with disease stage. We also obtained samples from age-matched healthy controls as a comparison. enzyme-linked immunosorbent assay (ELISA) showed that titin protein concentration in sera was significantly decreased in serum from patients of all EOC stages (I–IV) as compared to healthy individuals (Figure 3; *p* < 0.001). The mean titin protein concentration in healthy sera was 2,913.15 ± 406.73 pmol/L and was consistently decreased in advanced stages of OC, with titin protein concentrations of 1,661.95 ± 231.23, 1,132.00 ± 38.14, 861.13 ± 74.66, and 454.50 ± 91.01 pmol/L in stages I, II, III, and IV EOC patients, respectively (Figure 3).

### Titin is downregulated during acquisition of chemoresistance

**Figure 4:**
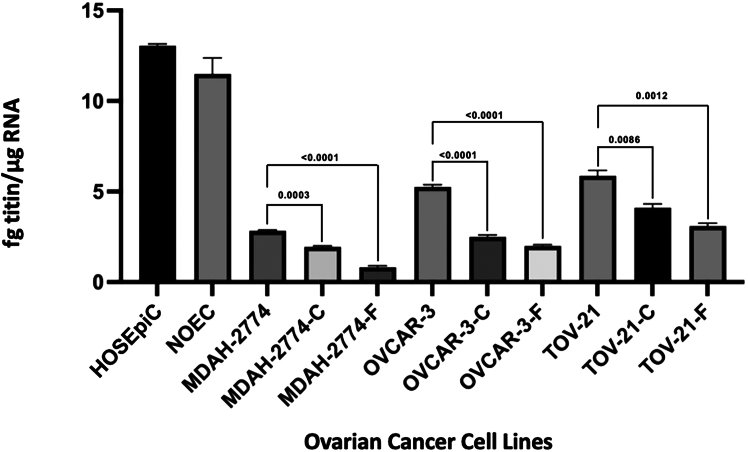
Titin expression in chemoresistant OC cells Quantitative real-time PCR was used to measure mRNA levels for titin in multiple chemosensitive and chemoresistant OC cell lines. Titin mRNA levels were determined in normal epithelial ovarian cell lines (HOSEpic, NOEC) and the chemosensitive epithelial OC cell lines and their cisplatin/docetaxel chemoresistant counterparts (MDAH-2774, OVCAR-3, TOV-21). Each quantitative real-time PCR was run in triplicate, with error bars showing the standard error.

Chemoresistance is one of the hallmarks of EOC progression. To determine whether the acquisition of chemoresistance is correlated with changes in titin expression, we generated cisplatin- and docetaxel-resistant cultures from three human EOC cell lines (MDAH-2774, OVCAR-3, and TOV-21). Cisplatin and docetaxel were chosen because they are accepted therapies for OC. Chemoresistant cell growth rate is very low as compared to chemosensitive cells; thus, enough protein to run an ELISA for titin protein level determination for all these samples was not possible. In our lab, we have developed real-time PCR as a sensitive technique that requires less material to accurately measure absolute mRNA levels for titin. When chemosensitive EOC cells were compared to chemoresistant counterparts, we consistently observed significantly lower levels of titin in the chemoresistant cells (Figure 4). In MDAH-2774 cells, titin mRNA levels were 2.83 ± 0.05 fg titin/μg RNA in chemosensitive cells, which decreased significantly to 1.94 ± 0.06 and 0.82 ± 0.09 fg titin/μg RNA in docetaxel- and cisplatin-resistant cells, respectively (*p* < 0.05). Similarly, OVCAR-3 cells exhibited higher titin mRNA levels at 5.25 ± 0.13 fg titin/μg RNA in chemosensitive cells, which decreased to 2.49 ± 0.12 and 1.99 ± 0.08 fg titin/μg RNA in cisplatin- and docetaxel-resistant cells, respectively (*p* < 0.05). In TOV-21G cells, titin mRNA levels were initially at 5.87 ± 0.3 fg/μg in chemosensitive cells, but they subsequently decreased to 4.11 ± 0.21 and 3.09 ± 0.16 fg titin/μg RNA in cisplatin- and docetaxel-resistant cells, respectively (*p* < 0.05; Figure 4).

Furthermore, the differences in titin expression were most pronounced in the MDAH-2774 cell line, with a decrease in titin mRNA from 1.94 ± 0.06 to 0.82 ± 0.09 fg titin/μg RNA between cisplatin- and docetaxel-resistant lines, respectively, thus highlighting a more significant decrease in titin expression in docetaxel-resistant cells. When comparing all EOC cell lines, the most significant change in titin expression was observed between chemosensitive and docetaxel-resistant MDAH-2774 cell lines, displaying a decrease from 2.83 ± 0.05 to 0.82 ± 0.09 fg titin/μg RNA. Overall, there was a more significant decrease in titin expression in docetaxel-resistant cells compared to cisplatin-resistant cells, consistent across the MDAH-2774, OVCAR-3, and TOV-21 cell lines (Figure 4). Finally, we determined titin levels in normal ovarian epithelial cell lines (human ovarian surface epithelial cells [HOSEpiC] and normal ovarian epithelial cells [NOEC] cells) and found titin mRNA levels to be higher relative to all EOC cell lines investigated (13.05 ± 0.10 and 11.49 ± 0.89 fg titin/μg RNA, respectively).

## Discussion

In this study, we show a significant decrease in titin expression in tissues and sera from advanced-stage OC compared to early-stage patients and healthy controls. Similarly, titin expression in EOC cells was downregulated relative to normal ovarian surface epithelial cells. Notably, titin exhibited lower expression levels in docetaxel- and cisplatin-resistant EOC cells compared to their chemosensitive counterparts. These findings illuminate the possible use of titin as a biomarker for EOC, opening avenues for further exploration.

Although titin protein is primarily associated with muscle function, it has emerged as a research target to better understand its role in other cellular processes and diseases.^9^^,^^12^^,^^17^^,^^18^ While the involvement of titin in cancer is not as extensively researched as in neuromuscular disorders, previous studies suggest its potential importance.^7^^,^^8^^,^^9^^,^^10^^,^^11^ Alterations in titin expression in breast, lung, colorectal, and gastrointestinal cancers have been strong indicators of its role in cancer development and progression.^7^^,^^8^^,^^9^^,^^10^^,^^11^

In colorectal cancer, a significant decrease in titin expression has been demonstrated.^7^ The study noted a strong association between titin expression and critical clinical factors such as clinical stage, node metastasis, histological type, race, and TP53 mutations.^7^ Intriguingly, higher titin expression was found to correlate with a lower likelihood of survival.^7^ Furthermore, downregulation of titin expression using titin-antisense RNA1 (titin-AS1) positively correlated with more advanced disease and poorer prognosis, such as tumor size, tissue invasion, and advanced stage in lung adenocarcinoma.^19^

Additionally, studies have reported changes in the expression of titin in breast cancer tissues that contributed to the invasive characteristics of breast cancer cells.^11^ Attenuated titin expression has also been reported to be associated with the progression and metastasis of lung and gastrointestinal cancers.^10^^,^^11^ However, it is important to note that the expression of titin in cancer may be influenced by various factors, including genetic mutations, epigenetic changes, and alterations in the tumor microenvironment. Collectively, these studies are consistent with the findings of the present study.

The role of titin in OC has not been extensively studied. However, others have investigated the role of the titin gene in OC and provided results consistent with ours.^17^^,^^20^ A previous study reported several mutations within the titin gene that could serve as genetic markers in identifying ovarian serous cystadenocarcinomas.^20^ The study found a positive correlation between the number of mutations in the titin gene and the number of mutations in the cancer cells. Moreover, the research examined the types of mutations observed in this gene, with the most prevalent being a missense mutation affecting the fibronectin type III and I-set immunoglobulin domains, suggesting a possible influence on angiogenesis within the tumor. Inhibition of titin expression by titin-AS1 altered the proliferation, invasion, and migration of OC cells *in vitro*.^21^ Furthermore, lung cancer patients with titin mutations also had a more favorable overall survival and a good chemotherapy response compared to patients with wild-type titin.^17^

In this study, we have shown the expression of titin to decrease in chemoresistant cell lines. We selected docetaxel and cisplatin because they are commonly used chemotherapeutic agents for treating OC. However, titin expression in chemoresistant patients could not be evaluated due to the lack of tissues and serum. To date, there are no other studies that have assessed titin expression with other chemotherapy treatments. Whether lower titin expression correlates with more resistance to chemotherapy remains an area for future investigation. Furthermore, a clear limitation of the study is the correlation of titin protein expression with prognosis, survival, and metastasis of OC. While it is important to correlate titin protein expression with prognosis, survival, and metastasis of OC, the diagnosis date and the date of death were not available for patients enrolled in this study. We are currently conducting a comprehensive analysis of a large cohort comprising 500 OC tumors, spanning stages I–IV and encompassing various histotypes. Our study incorporates extensive patient follow-up data and clinicopathological parameters. The primary objective is to investigate the correlation between titin expression and different stages, histotypes, and outcomes such as survival, treatment response, recurrence, and distant metastasis. Therapy for OC often involves a combination of surgery, chemotherapy, and, in some cases, targeted therapies. Several OC markers are available for diagnosis, prognosis, and guiding treatment decisions.

Our study acknowledges the inherent variability in sample sizes across different time periods, which is primarily attributed to the data available for serum analysis. It is important to note that this variation was carefully managed and validated by a professional biostatistician to ensure the integrity of our findings. However, we recognize that the observed variability in sample sizes poses a limitation to our study. Small sample sizes can impact the robustness and generalizability of our results. Despite meticulous statistical validation, the relatively limited sample sizes in certain periods may have influenced the precision of our estimates and introduced a degree of uncertainty. Future research endeavors could benefit from larger cohorts to further enhance the reliability of our findings.

Currently, there are several key markers for therapy in OC such as CA-125 (Cancer Antigen 125), HE4 (Human Epididymis Protein 4), BRCA1 and BRCA2 mutations, PD-L1 expression, MMR (Mismatch Repair) and MSI (Microsatellite Instability), HER2 (Human Epidermal Growth Factor Receptor 2), and CA-19-9. Exploring the downregulated expression of titin in OC holds potential preventive interventions, which may also lead to vaccine development for OC and potentially other cancers such as breast cancer. Exploring titin-based vaccine development for OC prevention holds significant promise as a future research avenue. Taking advantage of the immunogenic properties of titin to stimulate an immune response against OC cells, followed by rigorous evaluation in preclinical models, represents a novel and worthy endeavor. While the notion of employing titin in vaccine development for OC prevention presents exciting possibilities, comprehensive preclinical and clinical investigations are imperative to realize its potential as a viable therapeutic strategy. Furthermore, the integration of titin-based vaccination strategies with established OC prevention and treatment modalities presents an opportunity for synergistic benefits. The combination of titin-targeting vaccines with conventional therapies such as chemotherapy or immunotherapy may enhance therapeutic outcomes and mitigate disease recurrence.

## Materials and methods

### Human subjects

Tissues: the current study adhered to the Declaration of Helsinki and received approval from the Regional Ethical Review Board (case no. 767–14), which also granted a waiver of written consent for tumor specimen use. The cohort comprised primary invasive ovarian carcinoma patients diagnosed between 1994 and 2006. Tumor samples were sourced from the Sahlgrenska University Hospital Oncology lab in Gothenburg, Sweden, and reclassified by certified pathologists according to current World Health Organization criteria using corresponding formalin-fixed paraffin-embedded (FFPE) samples from the Department of Clinical Pathology at the same hospital. Neoplastic cell percentage was determined in all samples via May-Grünwald Giemsa-stained touch preparation imprints. Samples with at least 50% neoplastic cell content were selected for further analysis. Ovarian tumors were collected at the time of primary surgery.

Sera: serum samples were collected from patients diagnosed with OC of various stages and histology by the Gynecologic Oncology Division at the Karmanos Cancer Institute. Patients provided informed consent to provide blood samples prior to chemotherapy or surgery. The samples included all stages of diagnosis, regardless of the histotype. The sera samples included in this study were collected between 2012 and 2018, under Wayne State University Human Subject Committee protocol number 02-72-01(M02)-ER (approval date February 14, 2001). Patient demographics are summarized in Table 1.

### OC staging

OC staging is based on tumor growth and metastatic spread, with two primary staging systems: the International Federation of Gynecology and Obstetrics (FIGO) and the American Joint Committee on Cancer TNM (tumor, nodes, metastasis) system. The FIGO system, which is the most widely used, categorizes stages from I to IV: stage I denotes cancer restricted to the ovaries, stage II indicates spread to nearby pelvic organs, stage III shows extension to the abdomen or lymph nodes, and stage IV indicates distant metastasis.^19^^,^^22^^,^^23^ Staging criteria incorporate surgical findings, imaging, and pathology to evaluate tumor size, location, nearby tissue involvement, and distant metastases.

### Serum collection

Sera were collected from 32 EOC patients and 6 age-matched healthy female controls with no cancer history. Seven milliliters of blood were obtained from each subject and allowed to clot for 2 h prior to centrifugation at 2,400 rpm for 10 min. One-milliliter aliquots were stored at −80°C. Serum samples were deidentified and processed blindly.

### IHC

IHC was performed as previously described.^6^ FFPE blocks from 22 stage I, 17 stage II, 21 stage III, and 35 stage IV OC tissue samples were obtained from the Pathology Department at Wayne State University (MI, USA). Briefly, 4-μM FFPE sections were organized on Dako FLEX IHC microscope slides and dried at 60°C. The titin antibody (Santa Cruz [TX, USA], titin antibody [E−2], sc-271946) was optimized using a panel containing 10 full-face EOC specimens from various stages and histotypes. The titin antibody was raised against a common epitope at the C terminus of the *TTN* protein. In neoplastic cells, the staining intensity was blindly rated by a pathologist on a scale ranging from 0 to 3, with 0 indicating negative staining, 1 weak staining (low), 2 moderate staining (medium), and 3 strong staining (high). For each patient, the highest intensity value was used in the analyses.

### ELISA

Titin was quantified using a solid-phase sandwich ELISA (Human Titin N-Fragment [Serum] ELISA Kit, IBL-America, MN, USA) according to the manufacturer’s protocol. Briefly, the anti-titin-N (151A1) mouse immunoglobulin G (IgG) monoclonal antibody (which recognizes a common epitope in TTN protein) was coated on a plate. Samples or standards were then added to the wells for the first reaction. After the reaction, the horseradish peroxidase-conjugated secondary antibody (anti-titin-N [144A2] mouse IgG) was added to the wells for the second reaction. After washing unbound secondary antibody, tetramethyl benzidine was added. A standard (recombinant human titin, 1–200 dilution) was prepared by serial dilution (3,000, 1,500, 750, 375, 187.5, 93.75, and 46.88 pmol/L). The optical density of the standard and test samples was measured against a blank test sample at 450-nm wavelength.

### Cell culture

Normal human ovarian cell lines HOSEpiC and NOEC were obtained from ScienCell Research Laboratories (CA, USA) and Cell Biologics (IL, USA). Human OC cell lines SKOV-3, OVCAR-3, and TOV-21G were obtained from the American Type Culture Collection (VA, USA) and MDAH-2774 OC cells were obtained from, AcceGen (NJ, USA). Human primary ovarian cells included in this study were approved under Wayne State University institutional review board protocol number 2024-140 (approval date July 2024). The OC cell line A2780 and its cisplatin-resistant (1 μM) counterpart were obtained from Sigma-Aldrich (MO, USA). Docetaxel-resistant SKOV-3 and TOV-21G cell lines were established in-house by continuous culture in media containing a stepwise increase in either cisplatin (Sigma-Aldrich) or docetaxel (Sigma-Aldrich) over a period of 6 months, with a final concentration of 1.5 μM for cisplatin or 0.3 μM for docetaxel. We chose docetaxel and cisplatin because they constitute the most commonly used chemotherapy for the treatment of OC. Upon reaching final concentrations, the cells were grown in the absence of the chemotherapeutic drugs for 2 weeks, followed by replacement of the drug and verification of resistance by the trypan blue cell viability and MTT (3-(4,5-dimethylthiazol-2-yl)-2,5-diphenyltetrazolium bromide) cell proliferation assays. Doses were selected based on previously published studies.^24^^,^^25^

### Quantitative real-time PCR analysis

For the design of titin PCR primers and standard, one pair of universal *TTN* primers was selected through NCBI primer search on the human titin mRNA sequence (NM_0000012). Primers (Sigma-Aldrich) were designed to amplify a 73-bp fragment from the human *TTN* gene starting at nucleotide 3605. The primer sequences are as follows: forward 5′-CAGATCGATGGGTCCGTGTA-3′, reverse 5′-CCTTCAGTAAGGCCAGTGGA-3′. A standard sequence was synthesized (Sigma-Aldrich) to include the forward and reverse primer sequences, which also amplified a 73-bp product. The standard sequence is as follows: 5′-CAGATCGATGGGTCCGTGTAAATAAAGTACCTGTGA CAATGACACG GTACCGCTCCACTGGCCTTACTGAAGG-3′. Due to alternative splicing events in the titin gene, which give rise to at least five different isoforms for the human *TTN* gene, the *TTN* PCR primers were selected to amplify the first repeat region of the *TTN* gene. β-Actin (NM_001101) was used as the internal control with sequences: sense (5′-to-3′) ATGACTTAGTTGCGTTACAC and antisense (3′-to-5′) AATAAAGCCATGCCAATCTC, with a 79-bp standard flanked by the two primers. A standard curve was constructed by performing quantitative real-time PCR on stepwise dilutions of the standard to determine the concentration of *TTN* per microgram RNA in the samples, as previously described.^24^ The amount of mRNA was normalized to the abundance of the housekeeping gene β-actin.

Total RNA was isolated from cells utilizing the RNeasy Extraction Kit (Qiagen, MD, USA). cDNA synthesis was performed using the SuperScript VILO Master Mix Kit (Life Technologies, CA, USA). Quantitative real-time PCR was performed using a QuantiTect SYBR Green RT-PCR kit (Qiagen), the Mic qPCR Cycler. A standard with a known concentration was designed specifically for titin using the Beacon Designer software Free Edition (Premier Biosoft, CA, USA). This allowed for absolute quantification of gene expression as copy numbers per microgram of RNA. An initial melting cycle was performed at 95°C, followed by 40 cycles of denaturation at 95°C for 15 s, annealing at 64°C for 20 s, and an extension cycle at 72°C for 30 s. Following real-time PCR, a melting curve analysis was performed to demonstrate the specificity of the PCR product as a single peak. A control, which contained all the reaction components except for the template, was included in all experiments.

### Statistical analysis

Data are presented as mean ± SEM. Unpaired t test (for comparing two groups) or ordinary one-way ANOVA (for comparing more than two groups) was used to calculate statistical significance. *p* < 0.05 was considered significant. Data were graphed and analyzed using GraphPad Prism version 10.

## Data and code availability

All data were stored on the computer of the corresponding author’s main laboratory and can be requested from the corresponding author.

## Acknowledgments

The authors thank the Department of Obstetrics and Gynecology at Wayne State University School of Medicine. This work was supported in part by grant from the swedish cancer society (23 2732 Pi 01 H)

## Author contributions

Conceptualization: G.M.S.; data curation: G.M.S. and A.A.; formal analysis: G.M.S. and A.A.; funding acquisition: G.M.S.; methodology: G.M.S., H.S., A.A., A.N., J.A., and T.K.; project administration: G.M.S.; resources: G.M.S.; supervision: G.M.S.; writing – original draft: G.M.S.; writing – review & editing: R.T.M., G.M.S., H.S., A.A., A.N., J.A., A.B., T.Z.P., and K.H.

## Declaration of interests

The authors declare no competing interests.

## References

[1] Arora T., Mullangi S., Lekkala M.R. (2021). Ovarian cancer.

[2] Pokhriyal R., Hariprasad R., Kumar L., Hariprasad G. (2019). Chemotherapy resistance in advanced ovarian cancer patients. Biomarkers Cancer.

[3] Herzog W. (2018). The multiple roles of titin in muscle contraction and force production. Biophys. Rev..

[4] Krüger M., Linke W.A. (2011). The giant protein titin: a regulatory node that integrates myocyte signaling pathways. J. Biol. Chem..

[5] Mayans O., van der Ven P.F., Wilm M., Mues A., Young P., Fürst D.O., Wilmanns M., Gautel M. (1998). Structural basis for activation of the titin kinase domain during myofibrillogenesis. Nature.

[6] Nakanishi N., Tsutsumi R., Hara K., Matsuo M., Sakaue H., Oto J. (2021). Urinary titin N-fragment as a biomarker of muscle atrophy, intensive care unit-acquired weakness, and possible application for post-intensive care syndrome. J. Clin. Med..

[7] Wei H., Ren K., Zhang Q., Jin Y., Cao B., Tian Z., Mao T., Ren L. (2023). Titin as a potential novel therapeutic target in colorectal cancer. J. Cell Mol. Med..

[8] Xie X., Tang Y., Sheng J., Shu P., Zhu X., Cai X., Zhao C., Wang L., Huang X. (2021). Titin mutation is associated with tumor mutation burden and promotes antitumor immunity in lung squamous cell carcinoma. Front. Cell Dev. Biol..

[9] Su C., Wang X., Zhou J., Zhao J., Zhou F., Zhao G., Xu X., Zou X., Zhu B., Jia Q. (2021). Titin mutation in circulatory tumor DNA is associated with efficacy to immune checkpoint blockade in advanced non-small cell lung cancer. Transl. Lung Cancer Res..

[10] Yang Y., Zhang J., Chen Y., Xu R., Zhao Q., Guo W. (2020). MUC4, MUC16, and TTN genes mutation correlated with prognosis, and predicted tumor mutation burden and immunotherapy efficacy in gastric cancer and pan-cancer. Clin. Transl. Med..

[11] Sun E., Liu X., Lu C., Liu K. (2021). Long noncoding RNA TTNAS1 regulates the proliferation, invasion and migration of triplenegative breast cancer by targeting miR2115p. Mol. Med. Rep..

[12] Greenman C., Stephens P., Smith R., Dalgliesh G.L., Hunter C., Bignell G., Davies H., Teague J., Butler A., Stevens C. (2007). Patterns of somatic mutation in human cancer genomes. Nature.

[13] Oh J.H., Jang S.J., Kim J., Sohn I., Lee J.Y., Cho E.J., Chun S.M., Sung C.O. (2020). Spontaneous mutations in the single TTN gene represent high tumor mutation burden. NPJ Genom. Med..

[14] Zhu C., Guo W. (2017). Detection and quantification of the giant protein titin by SDS-agarose gel electrophoresis. MethodsX.

[15] (2024). TTN titin [Homo Sapiens (human)] - gene - NCBI. National Center for Biotechnology Information. https://www.ncbi.nlm.nih.gov/gene/7273.

[16] Tang Z., Li C., Kang B., Gao G., Li C., Zhang Z. (2017). GEPIA: a web server for cancer and normal gene expression profiling and interactive analyses. Nucleic Acids Res..

[17] Xue D., Lin H., Lin L., Wei Q., Yang S., Chen X. (2021). TTN/TP53 mutation might act as the predictor for chemotherapy response in lung adenocarcinoma and lung squamous carcinoma patients. Transl. Cancer Res..

[18] McCluggage W.G., Hirschowitz L., Ganesan R., Kehoe S., Nordin A. (2010). Which staging system to use for gynecological cancers: recommendations for practice in the United Kingdom. Int. J. Gynecol. Cancer.

[19] Jia Y., Duan Y., Liu T., Wang X., Lv W., Wang M., Wang J., Liu L. (2019). LncRNA TTN-AS1 promotes migration, invasion, and epithelial mesenchymal transition of lung adenocarcinoma via sponging miR-142-5p to regulate CDK5. Cell Death Dis..

[20] Gomes F.D.C., Figueiredo E.R.L., Araújo E.N.D., Andrade E.M.D., Carneiro C.D.L., Almeida G.M.D., Dias H.A.A.L., Teixeira L.I.B., Almeida M.T., Farias M.F.D. (2023). Social, Genetics and Histopathological Factors Related to Titin (TTN) Gene Mutation and Survival in Women with Ovarian Serous Cystadenocarcinoma: Bioinformatics Analysis. Genes.

[21] Miao S., Wang J., Xuan L., Liu X. (2020). LncRNA TTN-AS1 acts as sponge for miR-15b-5p to regulate FBXW7 expression in ovarian cancer. Biofactors.

[22] LeWinter M.M., Granzier H.L. (2013). Titin is a major human disease gene. Circulation.

[23] Matsuo M., Awano H., Maruyama N., Nishio H. (2019). Titin fragment in urine: A noninvasive biomarker of muscle degradation. Adv. Clin. Chem..

[24] Harper A.K., Kirsch-Mangu T.K., Lutfi H., Morris R.T., Saed G.M. (2023). Binding of Intracellular Myeloperoxidase to αV/β1 Integrin Serves as a Mechanism of Survival in Epithelial Ovarian Cancer. Reprod. Sci..

[25] Fletcher N.M., Belotte J., Saed M.G., Memaj I., Diamond M.P., Morris R.T., Saed G.M. (2017). Specific point mutations in key redox enzymes are associated with chemoresistance in epithelial ovarian cancer. Free Radic. Biol. Med..

